# Robot-assisted surgery and artificial intelligence-based tumour diagnostics: social preferences with a representative cross-sectional survey

**DOI:** 10.1186/s12911-024-02470-x

**Published:** 2024-03-27

**Authors:** Áron Hölgyesi, Zsombor Zrubka, László Gulácsi, Petra Baji, Tamás Haidegger, Miklós Kozlovszky, Miklós Weszl, Levente Kovács, Márta Péntek

**Affiliations:** 1 Doctoral School, Semmelweis University, Budapest, Hungary; 2 Health Economics Research Center, University Research and Innovation Center (EKIK), Óbuda University, Budapest, Hungary; 3 Musculoskeletal Research Unit, University of Bristol, Bristol, UK; 4 Antal Bejczy Center for Intelligent Robotics, University Research and Innovation Center (EKIK) , Óbuda University, Budapest, Hungary; 5 Austrian Center for Medical Innovation and Technology (ACMIT) , Wiener Neustadt, Austria; 6 BioTech Research Center, University Research and Innovation Center (EKIK) , Óbuda University, Budapest, Hungary; 7 John von Neumann Faculty of Informatics, Óbuda University, Budapest, Hungary; 8 Department of Translational Medicine, Semmelweis University, Budapest, Hungary; 9 Physiological Controls Research Center, University Research and Innovation Center (EKIK) , Óbuda University, Budapest, Hungary

**Keywords:** Public opinion, Social preference, Willingness to pay, Artificial intelligence, Robotic surgical procedures, Diagnostic imaging

## Abstract

**Background:**

The aim of this study was to assess social preferences for two different advanced digital health technologies and investigate the contextual dependency of the preferences.

**Methods:**

A cross-sectional online survey was performed among the general population of Hungary aged 40 years and over. Participants were asked to imagine that they needed a total hip replacement surgery and to indicate whether they would prefer a traditional or a robot-assisted (RA) hip surgery. To better understand preferences for the chosen method, the willingness to pay (WTP) method was used. The same assessment was conducted for preferences between a radiologist’s and AI-based image analysis in establishing the radiological diagnosis of a suspected tumour. Respondents’ electronic health literacy was assessed with the eHEALS questionnaire. Descriptive methods were used to assess sample characteristics and differences between subgroups. Associations were investigated with correlation analysis and multiple linear regressions.

**Results:**

Altogether, 1400 individuals (53.7% female) with a mean age of 58.3 (SD = 11.1) years filled in the survey. RA hip surgery was chosen by 762 (54.4%) respondents, but only 470 (33.6%) chose AI-based medical image evaluation. Those who opted for the digital technology had significantly higher educational levels and electronic health literacy (eHEALS). The majority of respondents were willing to pay to secure their preferred surgical (surgeon 67.2%, robot-assisted: 68.8%) and image assessment (radiologist: 70.9%; AI: 77.4%) methods, reporting similar average amounts in the first (*p* = 0.677), and a significantly higher average amount for radiologist vs. AI in the second task (*p* = 0.001). The regression showed a significant association between WTP and income, and in the hip surgery task, it also revealed an association with the type of intervention chosen.

**Conclusions:**

Individuals with higher education levels seem to accept the advanced digital medical technologies more. However, the greater openness for RA surgery than for AI image assessment highlights that social preferences may depend considerably on the medical situation and the type of advanced digital technology. WTP results suggest rather firm preferences in the great majority of the cases. Determinants of preferences and real-world choices of affected patients should be further investigated in future studies.

**Supplementary Information:**

The online version contains supplementary material available at 10.1186/s12911-024-02470-x.

## Background

As a result of the technological changes of the past few decades, healthcare has seen an increasing uptake of digital technologies, including robotics, artificial intelligence (AI) and machine learning [1–3]. Fast and efficient data handling, reduced workload, option of remote control with physical separation and improved accuracy are just some of the key factors that have played a major role in their adoption for various functions, including administrative tasks, data processing, telemedicine and patient education [4, 5]. 

One of the main areas of application is diagnostic imaging, where AI-based methods have shown promising results in terms of accuracy, sensitivity and specificity in the segmentation and interpretation of radiological images [6–8]. The use of AI in the clinical environment, however, is not without its limitations. The most common challenges include the need for time-consuming and resource-intensive user training, high hardware requirements, insufficient integration into the clinical workflow, ethical and legal implications, and the lack of or limited transparency of operation, which can lead to uncertainty about the accuracy and reliability of the results [9, 10]. 

Another area of focus is surgery, where the assistance of robots might offer numerous advantages over conventional methods, including increased accuracy, better implant positioning and improved radiological outcomes, as well as ergonomic benefits and reduced workload for surgeons [11–14]. However, some concerns have also been raised, such as the scarcity of long-term follow-up data and uncertain results on patient outcomes such as functioning, quality of life and perceived pain levels [12, 15]. Furthermore, several studies failed to prove improvements in complication rates compared with conventional methods [16]. 

While most clinicians are aware of the existence of new advanced digital health technologies, they have limited real-world experience, evidence-based knowledge and well-established clinical guidelines and, therefore, remain unconvinced about the reliability and accuracy of the results [17]. This can be a barrier to the adoption of new technologies in clinical practice, as acceptance and learning of their use by healthcare professionals play a key role in the process [18]. However, patient attitudes towards complex digital health technologies may also have a significant impact on their implementation. Patient informed consent is essential and also clinicians are more likely to have positive attitudes and adopt technologies that are better accepted by their patients [19]. A number of factors have been shown to improve patient acceptance of advanced digital health technologies, such as use in lower-risk conditions, proven higher accuracy compared to human professionals, or even when the technology is recommended or preferred by the treating physician or healthcare provider [20–24]. 

Despite their increasing adoption and use, there is a scarcity of studies reporting on patients’ perspectives and outcomes, as well as on social attitudes and preferences towards advanced digital health technologies [15, 25]. Exploring preferences of the society is relevant as, on the one hand, it includes the potential target patients and the social environment (e.g., patients’ family members, acquaintances) that might influence their health-related decisions. On the other hand, while we acknowledge that societal acceptance of a new health technology can be driven by a broad range of factors besides evidence on health outcomes, revealing social preferences can give an approximate idea about the expected societal endorsement of their financing decisions.

Willingness to pay (WTP) is a valuation method that allows for assessing social preferences for a diverse range of products and services, including health technologies [26, 27]. In healthcare, measuring WTP is based on the assumption that the value and benefits of a given health technology can be determined by examining the ability to make trade-offs between the consumption of goods and factors that may improve health [28]. WTP is particularly well suited to measuring the values and benefits of technologies that have a multifaceted nature [29, 30]. A recent systematic review identified studies that used alternative valuation methods, including WTP, to examine the socio-economic and health benefits of medical devices, non-device health technologies and methodologies [31]. In the majority of studies, the WTP valuation method was used. Although, only one study evaluated a digital technology (robotic radiosurgery), and despite the digital developments in orthopedics, rheumatology or radiological image analysis, no study was found that assessed a digital technology in these segments [31]. 

Therefore, given the limited number of preference elicitation studies, there is a need to evaluate advanced digital health technologies from a broader perspective and to provide information on social preferences for their uses. This information would be of particular interest to clinicians (shared decision-making), researchers and developers (to guide future development directions and design clinical interventions), and health policymakers. It might also be relevant from a public health perspective, as it can drive the attention to subgroups that have reservations towards complex digital health technologies, thus patient education is particularly important in their case. The primary aim of this study was to assess social preferences for the use of different advanced digital health technologies in surgery and diagnostic imaging, as well as to examine the strength of preferences using WTP method. The secondary objective was to investigate how these preferences and WTP are associated with socio-demographic characteristics, health status and electronic health literacy. The results obtained with the two types of advanced technology are compared indirectly.

## Methods

### Study description

The present study was part of a larger survey on the knowledge about and attitudes towards implantable medical devices in the Hungarian population, details have been reported elsewhere [32]. The online cross-sectional study was conducted in July of 2021, involving a sample of the Hungarian general population aged 40 years and over. Quota sampling was applied to ensure the representativeness of the sample for sex, age, education and type of residence. Data collection was carried out by a survey company, participants were recruited from a commercial online panel. Dropout rates and the size of the sampling frame was confidential information, and have not been released by the survey company. The targeted sample size was 1400 respondents. Ethical approval was granted by the Hungarian Medical Research Council (no. IV/5651-1/2021/EKU). Respondents were informed that participation in the survey was voluntary, that their data would remain anonymous and would be used for scientific purposes only. Participants provided written informed consent before the start of the survey.

### The questionnaire

The survey consisted of three modules: (1) the epidemiology of and patients’ knowledge about implantable medical devices (IMDs) [32]; (2) subjective preferences for robot-assisted (RA) hip replacement surgery and AI-based assessment of a preoperative imaging scan; (3) subjective expectations for having IMDs at older ages. In this paper, results of the second module are presented. Survey questions translated into English are presented in Online Resource 1.

The socio-demographic characteristics of the sample, such as respondents’ sex, age, educational level, residency, family status and working status, were surveyed. Monthly net household income was recorded in 11 predefined categories, increasing equally with 140 EUR in each category, starting from 0 to 140 EUR and ending at 1260–1400 EUR in the 10th category. It was possible to indicate in an 11th category if the monthly net household income exceeded 1400 EUR.

In addition, electronic health literacy and general health state were surveyed with the eHEALS and the EQ-5D-5 L measurement tools, respectively. A detailed description of these outcome measures is provided below. A predefined list of IMDs was used to survey whether the respondent ever had or has an IMD.

### Measurement tools

#### Electronic Health Literacy Scale (eHEALS)

The eHEALS was developed to measure respondents’ self-assessed knowledge, confidence, and ability to find, understand and use health-related electronic information resources [33]. The tool consists of 8 questions that can be rated on a 5-point scale (1 - ‘strongly disagree’; 5 - ‘strongly agree’). To calculate the total score, the points for each question are summarized, resulting in a final score of 8 to 40. Higher score indicates higher e-Health literacy. In the present study, the validated Hungarian version of the eHEALS was used [34]. 

#### EQ-5D-5 L

The EQ-5D-5 L questionnaire measures respondents’ health in five dimensions: mobility, self-care, usual activities, pain and discomfort, anxiety and depression [35]. Respondents are asked to indicate what best describes their actual state in each dimension on a 5-level Likert scale (response options: 1 - no problems, 2 - slight problems, 3 - moderate problems, 4 - severe problems, 5 - unable to /extreme problems). Given all the possible combinations, there are 3125 health states that can be distinguished with the EQ-5D-5 L. The EQ-5D-5 L index score can be calculated by attaching preference-based scores, i.e. utility values to these states. In this study, the EQ-5D-5 L value set for Hungary was used [36]. The EQ VAS, as the second part of the EQ-5D-5 L, measures respondents’ actual self-reported overall health on a visual analogue scale, ranging from 0 to 100, indicating the worst and best health states the respondent can imagine.

#### Stated preferences and willingness to pay for hip replacement

Respondents were put into two hypothetical decision-making situations. In the first task, they had to imagine that they needed hip replacement surgery due to a gradually developing disease that limited their everyday activities. It was explained that a surgical robot has been developed that is able to perform some phases of the operation completely autonomously. In the case of an adverse event, the doctor could still switch off the robot at any time, and take over the operation. The traditional and the RA procedures were described as equally safe and produce the same results per outcome. Participants were asked to choose which method they would have preferred: a surgery performed primarily by a human surgeon (conventional surgery) or an RA surgery.

Next, each respondent was assigned to the intervention with the opposite method to the one they had chosen. Respondents were asked how much money they would be willing to pay to have the operation made by the preferred method chosen by the respondent in the previous question. Willingness to pay was recorded in the following 9 categories: 0 EUR (representing no willingness to pay); 0–28 EUR; 28–84 EUR; 84–140 EUR; 140–280 EUR; 280–560 EUR; 560–1120 EUR; 1120–2240 EUR; 2240 < EUR. For the highest category (2240 < EUR), respondents were asked to indicate the amount of money they were willing to pay. (The upper price range was set to be close to the market price at the time of the questionnaire survey.)

#### Stated preferences and willingness to pay for radiological image assessment task

In the second task, respondents were presented with a scenario in which a mandatory imaging scan prior to the hip replacement surgery revealed a suspected tumour. The treatment depends on whether the tumour is benign or malignant. They were asked whether they preferred a radiologist to analyse the image and establish the diagnosis or an AI, i.e., a computer algorithm trained to make a diagnosis based on the analysis of thousands of similar cases.

Respondents were then informed that the assessment had been carried out with the contrary method (but no information was provided about the result) and were asked how much money they would be willing to pay in order to obtain a secondary expert opinion with their preferred method. The following 9 categories, based on the market price of the interventions at the time of the survey, were used to record willingness to pay: 0 EUR (representing no willingness to pay); 0–3 EUR; 3–14 EUR; 14–42 EUR; 42–70 EUR; 70–98 EUR; 98–280 EUR; 280–839 EUR; 839 < EUR. For the highest category (839 < EUR), respondents were asked to indicate the amount of money they were willing to pay.

#### Respondents’ assessment of the difficulty of the tasks

After each task, respondents were asked to rate their level of agreement with ’Questions about hip replacement surgery were difficult to answer’ and ’It was difficult to answer the questions about the evaluation of the images’, on a 7-level scale (1: totally agree; 4: neither agree nor disagree; 7: totally disagree). Furthermore, participants who reported any level of difficulty also had to indicate the reason why they found it difficult to answer the questions using the following response options: because it was difficult to understand the situation caused by the outlined condition, imagine the need for hip replacement/the suspicion of having a tumour, understand the two medical procedures, choose between the two medical procedures, or indicate the amount they would be willing to pay. If participants found it more suitable they could also provide free-text responses as ’Other’ category.

### Statistical analysis

Background factors (socio-demographic characteristics, health status, electronic health literacy), respondents’ choices and WTP were analyzed with descriptive statistical methods. Differences by subgroups were tested with Chi-square, Mann-Whitney U, and Kruskal-Wallis tests for categorical variables, and with two sample t-tests for continuous ones.

Respondents’ income and WTP were recorded in Hungarian forint and converted subsequently to Euro for the analysis. The used exchange rate was 357.49 HUF/EUR.

In the survey, we included response options ’Do not know’ and ’Do not want to answer’ for the income-related questions. Such responses were treated as missing values and were excluded from the analysis.

Monthly net income per capita was calculated by dividing the middle point of each income category by the number of household members. The method proposed by Parker and Fenwick was used to determine the mean value of the top income category [37]. Furthermore, respondents were divided into 5 groups based on their monthly net income per capita, reflecting which national income quintile they belong to. The second, third, fourth and fifth quintiles were calculated from the average of the third to eighth national income deciles given by the Hungarian Central Statistical Office [38]. 

WTP was converted and treated as a continuous variable by assigning the middle value of the corresponding category to each respondent. In the highest category, the exact values provided by the respondents were used.

The correlation of willingness to pay with background variables was investigated by calculating Pearson’s correlation. The correlation was considered strong over 0.5, moderate between 0.5 and 0.3, and weak under 0.3 [39]. The normality of continuous variables was examined with the Shapiro-Wilk test [40]. 

In the subgroup comparison, the effect size was measured with Cohen’s D (small = 0.2; medium = 0.5; large = 0.8) [41]. 

Multiple linear regression analysis was carried out to assess which factors are associated with the respondents’ WTP. Two separate regression models were developed for the two WTP tasks. In both models, the dependent variable was the amount of money offered by the respondent in the respective WTP task. The independent variables were the preferred method chosen for hip replacement surgery (Model 1) and radiological image assessment (Model 2). In addition, both models were controlled for socio-demographic variables (sex, age, education, health education, residence, employment status, marital status, living with someone in the household), income, eHEALS score, EQ-5D-5 L index, whether the respondent had an implant, and level of difficulty at answering the WTP task questions. All categorical variables were dummy-coded before the analysis. The constant term was excluded from the analysis in both models.

The significance level of 0.05 was applied for all statistical tests.

Statistical analysis was performed in Stata 17 software (StataCorp LCC., College Station, TX, USA).

## Results

### Sample characteristics

Altogether, 1400 respondents completed the survey. Main characteristics of the sample are summarized in Table 1. The average age was 58.3 years (SD = 11.1) and 53.7% were women. Among the 584 participants (41.7%) who ever had at least one implant surgery, 33 reported having had a hip implant, and 32 were still living with that at the time of the survey. The average eHEALS score was 28.1 (SD = 5.8) on the 8–40 scale (men: 27.9, SD = 5.9; women: 28.3, SD = 5.6). The average EQ-5D-5 L index score was 0.83 (SD = 0.26) and EQ VAS was 75.1 (SD = 19.9).

**Table 1:** Characteristics of the sample

	Total sample	Preferred method of surgery		Preferred method for the radiological image assessment	
		Surgeon	Robot-assisted	Radiologist	AI
Variables	***N (%)***	***N (%)***		***N (%)***	
**Total**	1400 (*100)*	638 (*100)*	762 (*100)*	930 (*100)*	470 (*100)*
**Sex**		*p* = 0.001^**a**^		*p* = 0.193^a^	
*Men*	648 (*46.3)*	263 (*41.2)*	385 (*50.5)*	419 *(45.0)*	229 *(48.7)*
*Women*	752 (*53.7)*	375 (*58.8)*	377 (*49.5)*	511 *(55.0)*	241 *(51.3)*
**Age group, years**		*p* = 0.101^b^		*p* < 0.001^**b**^	
*40–44*	190 (*13.6)*	99 (*15.5)*	91 (*11.9)*	141 *(15.2)*	49 *(10.4)*
*45–49*	188 (*13.4)*	88 (*13.8)*	100 (*13.2)*	134 (*14.5)*	54 (*11.5)*
*50–54*	163 (*11.6)*	74 (*11.6)*	89 (*11.7)*	108 (*11.6)*	55 (*11.7)*
*55–59*	198 (*14.1)*	95 (*14.9)*	103 (*13.5)*	133 (*14.3)*	65 (*13.9)*
*60–64*	227 (*16.2)*	87 (*13.6)*	140 (*18.4)*	149 (*16.0)*	78 (*16.7)*
*65–69*	182 (*13.0)*	83 (*13.0)*	99 (*13.0)*	117 (*12.6)*	65 (*13.8)*
*70–74*	127 (*9.1)*	52 (*8.2)*	75 (*9.8)*	72 (*7.7)*	55 (*11.7)*
*75+*	125 (*8.9)*	60 (*9.4)*	65 (*8.5)*	76 (*8.2)*	49 (*10.4)*
**Education**		*p* < 0.001^**b**^		*p* = 0.048^**b**^	
*Primary*	410 (*29.3)*	228 (*35.7)*	182 (*23.9)*	281 (*30.2)*	129 (*27.4)*
*Secondary*	533 (*38.1)*	244 (*38.2)*	289 (*37.9)*	364 (*39.1)*	169 (*36.0)*
*Tertiary*	457 (*32.6)*	166 (*26.0)*	291 (*38.2)*	285 (*30.7)*	172 (*36.6)*
**Health education**		*p* = 0.213^a^		*p* = 0.458^a^	
*Yes*	103 (*7.4)*	53 (*8.3)*	50 (*6.6)*	65 (*7.0)*	38 (*8.1)*
*No*	1297 (*92.6)*	585 (*91.8)*	712 (*93.4)*	865 (*93.0)*	432 (*91.9)*
**Settlement type**		*p* = 0.020^**b**^		*p* = 0.357^b^	
*Capital*	315 (*22.5)*	131 (*20.5)*	184 (*24.1)*	207 (*22.3)*	108 (*23.0)*
*Town*	749 (*53.5)*	337 (*52.8)*	412 (*54.1)*	491 (*52.8)*	258 (*54.9)*
*Village*	336 (*24.0)*	170 (*26.7)*	166 (*21.8)*	232 (*24.9)*	104 (*22.1)*
**Married/having a partner**		*p* = 0.928^a^		*p* = 0.141^a^	
*Yes*	854 (*61.0)*	390 (*61.1)*	464 (*60.9)*	580 (*62.4)*	274 (*58.3)*
*No*	546 (*39.0)*	248 (*38.9)*	298 (*39.1)*	350 (*37.6)*	196 (*41.7)*
**Living with someone in the household**		*p* = 0.540^a^		*p* = 0.525^a^	
*Yes*	1064 (*76.0)*	480 (*75.2)*	584 (*76.6)*	702 (*75.5)*	362 (*77.0)*
*No*	336 (*24.0)*	158 (*24.8)*	178 (*23.4)*	228 (*24.5)*	108 (*23.0)*
**Paid work (missing = 30)**		*p* = 0.451^a^		*p* = 0.471^a^	
*Yes*	1287 (*91.9)*	581 (*91.1)*	706 (*92.7)*	856 (*92.0)*	431 (*91.7)*
*No*	83 (*5.9)*	41 (*6.4)*	42 (*5.5)*	52 (*5.6)*	31 (*6.6)*
**Household income category** ^**d**^ **(missing = 217)**		*p* = 0.001^**b**^		*p* = 0.063^b^	
*1st quintile*	261 (*18.6)*	144 (*22.6)*	117 (*15.4)*	180 *(19.4)*	81 *(17.2)*
*2nd quintile*	224 (*16.0)*	107 (*16.8)*	117 (*15.4)*	143 *(15.4)*	81 *(17.2)*
*3rd quintile*	237 (*16.9)*	102 (*16.0)*	135 (*17.7)*	171 *(18.4)*	66 *(14.0)*
*4th quintile*	201 (*14.4)*	84 (*13.2)*	117 (*15.4)*	126 *(13.6)*	75 *(16.0)*
*5th quintile*	260 (*18.6)*	98 (*15.4)*	162 (*21.4)*	158 *(17.0)*	102 *(21.7)*
**Any implant ever**		*p* = 0.237^a^		*p* = 0.008^**a**^	
*Yes*	584 (*41.7)*	277 (*43.4)*	307 (*40.3)*	365 (*39.3)*	219 (*46.6)*
*No*	816 (*58.3)*	361 (*56.6)*	455 (*59.7)*	565 (*60.7)*	251 (*53.4)*
**eHEALS score; mean (SD)**		*p* = 0.003^**c**^		*p* = 0.010^**c**^	
	28.1 (*5.8)*	27.6 (*6.0)*	28.5 (*5.5)*	27.8 *(5.7)*	28.6 *(5.5)*
**EQ-5D-5 L index score; mean (SD)**		*p* = 0.070^c^		*p* = 0.372^c^	
	0.83 (0.26)	0.82 (0.28))	0.84 (0.25)	0.83 (0.27)	0.84 (0.25)
**EQ VAS; mean (SD)**		*p* = 0.060^c^		*p* = 0.699^c^	
	75.1 (19.9)	74.0 (20.5)	76.0 (19.3)	74.9 (20.3)	75.4 (19.1)

Differences in the values of binary, ordinal and continuous variables were compared with Chi-square^a^, Mann-Whitney U^b^ and two sample t-tests^c^, respectively

^d^ ’Do not know’ and ’Do not want to answer’ responses were treated as missing values and excluded from the analysis

### Stated preferences for hip replacement surgery and radiological image evaluation

In the hip replacement task, 762 (54.4%) respondents preferred the robot-assisted (RA) surgery. Analysis by patient characteristics revealed that respondents’ preference was significantly associated with sex as compared to the women-men ratio observed in the total sample (53.7% vs. 46.3% respectively) the proportion of women was higher for the conventional (58.8%) and lower for the RA method (49.5%). (Table 1) The two subgroups did not differ significantly by age (means: 57.9 years, SD = 11.4 for the conventional and 58.7 years, SD = 10.8 for the RA surgery, *p* = 0.21). The average net income per capita was 378.2 (SD = 9.3) EUR and 446.3 (10.0) EUR in the subgroups choosing the conventional and the RA hip replacement surgery, respectively. The two subgroups differed significantly by income quintile group (individuals with higher income are more likely to choose the RA surgery). No significant difference was found between subgroups based on whether or not they had any IMD in the respondents’ history.

In the image evaluation task 470 (33.6%) respondents chose the AI-based image assessment. Respondents who preferred to have their radiological image analysed by AI were significantly older than those who chose the radiologist (means: 59.8 years, SD = 10.9 vs. 57.6 years, SD = 11.1, respectively; *p* < 0.05). The average net income per capita was 403.7 (SD = 8.6) EUR and 438.1 (SD = 11.8) EUR among those who chose the radiologist or the AI to make the diagnosis, respectively. However, no significant difference was found by income quintile groups. Respondents who had IMD were more likely to choose the AI-based image assessment.

In both tasks, respondents who opted for the digital health technology had significantly higher levels of education compared to those who opted for the conventional method. However, there was no difference according to whether respondents had any degree in health education. Those who chose the conventional method had significantly lower eHEALS scores compared to those who chose the digital technology both in the hip replacement surgery and the radiological image assessment task, although the observed differences were considered small as measured with the Cohen’s D (D=-0.159, 95% CI -0.264 - -0.053 and D=-0.145, 95% CI -0.256 - -0.034, respectively). No significant differences were observed in respondents’ health status as measured by the EQ-5D-5 L index score and EQ VAS in the two groups.

There was a great amount of respondents in the total sample who chose the physician (surgeon or radiologist; *N* = 504, 36.0%) for both tasks, their mean age was 57.4 (SD = 11.4) years, 58.1% of them were women. Fewer respondents chose the advanced digital technology (*N* = 336, 24.0%) in both cases, they were slightly older with a mean age of 59.6 (SD = 10.9) years and there were fewer women (47.3%) among them.

### Willingness to pay for hip replacement surgery

In the hip replacement surgery task, about one-third of participants were not willing to pay to have the intervention performed with the method of their preferred choice. The maximum amount offered was 5315 EUR for the conventional and 2797 EUR for the RA surgery. (Table 2.)

**Table 2:** Distribution of participants across willingness to pay categories

Preferred method of surgery			Preferred method for the radiological image assessment		
**WTP categories** (EUR; range)	**Surgeon** (N = 637)*	***Robot-assisted (N = 762)***	**WTP categories** (EUR; mean)	Radiologist (N = 928)*	**AI** ***(N = 470)***
	***N (%)***	***N (%)***		***N (%)***	***N (%)***
0**	209(32.8)	238 (31.2)	0**	271 (29.2)	106 (22.5)
**0–28**	80 (12.6)	65 (8.5)	**0–3**	62 (6.7)	19 (4.0)
**28–84**	73 (11.5)	100(13.1)	**3–14**	82 (8.8)	43 (9.1)
**84–140**	91 (14.3)	117 (15.4)	**14–42**	163 (17.6)	78 (16.6)
**140–280**	94 (14.8)	120 (15.8)	**42–70**	114 (12.3)	56 (11.9)
**280–560**	53 (8.3)	74 (9.7)	**70–98**	97 (10.5)	70 (14.9)
**560–1120**	19 (3.0)	33 (4.3)	**98–280**	107 (11.5)	73 (15.5)
**1120–2240**	12 (1.9)	14 (1.8)	**280–839**	32 (3.5)	25 (5.3)
**> 2240**	6 (1.0)	1 (0.1)	**> 839**	0 (0)	0 (0)

Conversion: 1 EUR = 357.49 HUF

*There was a missing value for one respondent

**The 0 category represents no willingness to pay

The average amount of money that the respondents were willing to pay did not differ significantly between respondents who chose conventional or RA surgeries. In both subgroups, significant differences were observed by educational level and income groups. (Table 3.; Online resource 2)

**Table 3:** Willingness to pay by socio-demographic subgroups

	Preferred method of surgery		Preferred method for the radiological image assessment	
Variables	Surgeon (*N* = 638)	Robot-assisted (*N* = 762)	Radiologist (*N* = 930)	AI (*N* = 470)
	EUR; mean (SD)			
**Total**	*p* = 0.153		*p* < 0.001	
	178.5 (429.5)	170.4 (300.0)	62.5 (110.8)	83.7 (129.5)
**Sex**	*p* = 0.311	*p* = 0.485	*p* = 0.922	*p* = 0.896
*Men*	168.4 (405.7)	167.8 (268.9)	70.2 (123.8)	81.4 (122.0)
*Women*	185.6 (445.9)	173.0 (329.0)	56.1 (98.6)	85.9 (136.5)
**Age group, years**	*p* = 0.001	*p* = 0.085	*p* = 0.161	*p* = 0.112
*40–44*	115.3 (228.7)	184.1 (293.6)	50.0 (91.1)	69.8 (114.2)
*45–49*	168.3 (395.8)	143.5 (247.4)	63.2 (113.7)	63.4 (98.0)
*50–54*	103.4 (228.1)	114.6 (214.4)	46.6 (89.3)	91.4 (158.0)
*55–59*	145.9 (372.9)	145.7 (282.5)	55.6 (96.1)	78.6 (138.1)
*60–64*	189.4 (334.7)	177.4 (306.0)	71.2 (119.0)	84.2 (130.1)
*65–69*	156.5 (275.6)	160.2 (244.7)	52.0 (63.4)	70.3 (87.0)
*70–74*	317.8 (759.9)	219.3 (374.1)	74.5 (147.0)	106.9 (160.7)
*75+*	338.2 (753.5)	251.5 (436.5)	106.3 (168.3)	109.4 (134.5)
**Education**	*p* = 0.013	*p* = 0.026	*p* < 0.001	*p* = 0.004
*Primary*	124.2 (290.1)	146.3 (288.0)	49.6 (106.9)	60.8 (113.2)
*Secondary*	181.4 (478.3)	144.2 (232.5)	55.6 (91.2)	83.5 (129.7)
*Tertiary*	249.3 (500.9)	211.4 (357.7)	83.9 (132.7)	101.1 (138.5)
**Health education**	*p* = 0.531	*p* = 0.300	*p* = 0.782	*p* = 0.514
*Yes*	272.3 (653.4)	210.9 (330.3)	77.9 (148.2)	102.8 (151.2)
*No*	170.0 (402.9)	167.5 (297.8)	61.3 (107.5)	82.0 (127.5)
**Settlement type**	*p* = 0.832	*p* = 0.028	*p* = 0.540	*p* = 0.024
*Capital*	198.5 (406.9)	204.1 (310.9)	61.5 (99.8)	94.4 (123.3)
*Town*	193.9 (500.4)	178.2 (327.7)	65.7 (117.9)	85.3 (136.6)
*Village*	132.7 (260.1)	113.4 (188.9)	56.5 (104.6)	68.6 (116.7)
**Household income category** ^**a (**^ **missing = 217)**	*p* < 0.001	*p* < 0.001	*p* < 0.001	*p* < 0.001
*1st quintile*	117.2 (288.2)	83.8 (195.9)	48.0 (106.9)	45.6 (97.6)
*2nd quintile*	79.5 (118.9)	121.5 (219.3)	40.8 (78.3)	62.6 (114.0)
*3rd quintile*	249.8 (640.8)	176.7 (313.5)	65.3 (112.2)	96.8 (147.7)
*4th quintile*	217.3 (330.9)	174.4 (252.8)	71.9 (107.9)	92.3 (117.2)
*5th quintile*	311.4 (666.8)	239.2 (381.0)	97.3 (148.9)	102.4 (124.0)
**Any implant ever**	*p* = 0.058	*p* = 0.058	*p* = 0.375	*p* < 0.001
*Yes*	200.3 (455.3)	188.4 (311.3)	65.1 (110.2)	96.8 (133.6)
*No*	161.8 (408.6)	158.2 (291.9)	60.8 (111.2)	72.3 (125.0)

Differences in WTP were tested with Kruskal-Wallis tests

^a^ ’Do not know’ and ’Do not want to answer’ responses were treated as missing values and excluded from the analysis

**Fig. 1:**
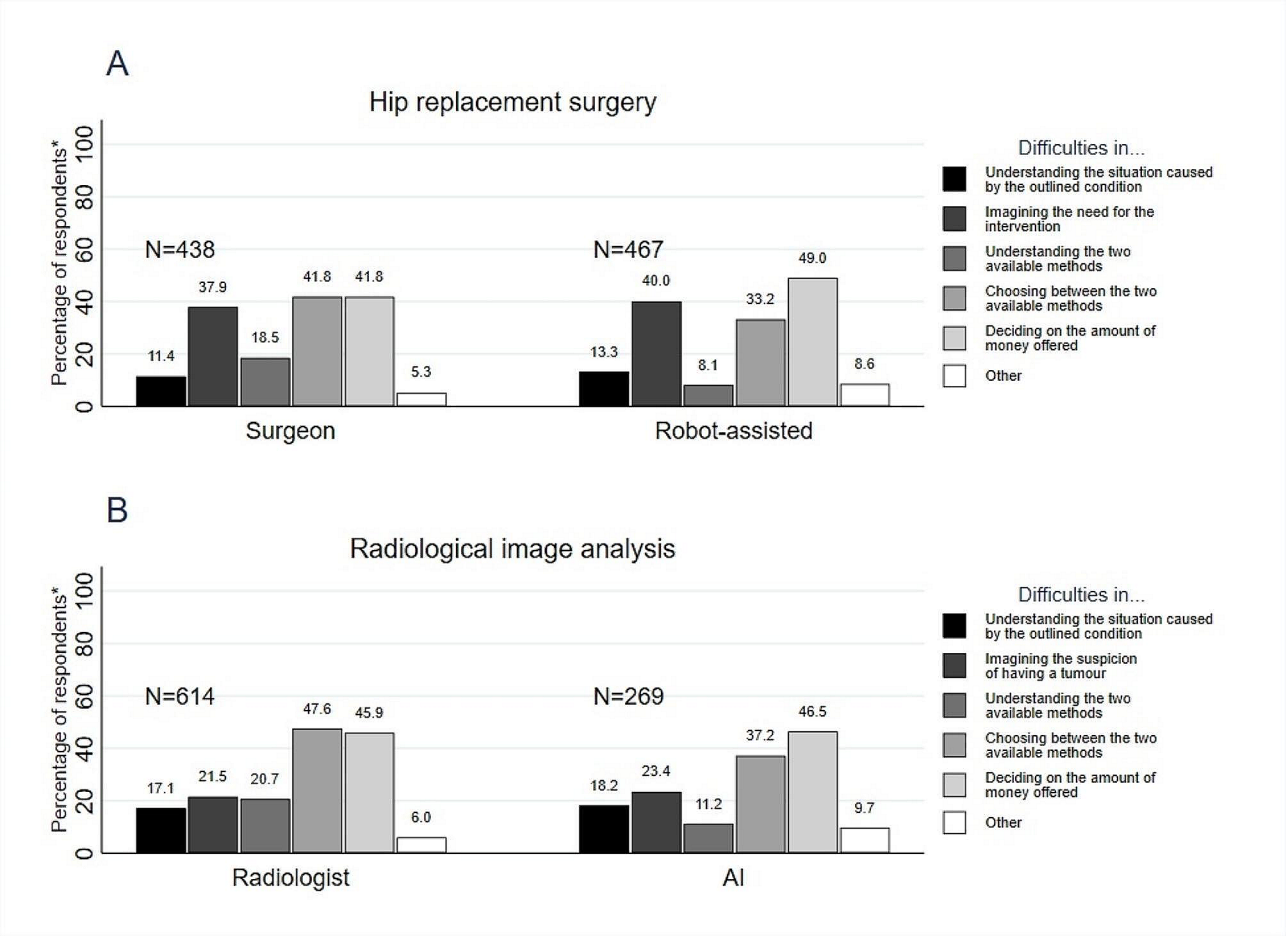
Frequency of reasons why respondents found questions difficult to answer in the two WTP tasks * Percentages refer to the proportion of a given reason among respondents who reported at least one difficulty. Percentages do not add up to 100% as respondents could indicate more than one difficulty

More than a third of respondents totally disagreed that questions about hip replacement were difficult to understand (31.4% in the conventional and 38.7% in the RA surgery subgroups), and a similar proportion was neutral (neither agreed nor disagreed) in the conventional surgery subgroup (33.4%), but just over a fifth in the RA surgery subgroup (22.7%). (Online resource 3.) For those who reported difficulties with at least one of the pre-defined response options in the surgeon (*N* = 438) and the RA surgery (*N* = 467) subgroups, the most commonly indicated problems were deciding on the amount of money offered (41.8% and 49.0%, respectively), choosing between the two methods (41.8% and 33.2%, respectively), and imagining the need for the intervention (37.9% and 40.0%, respectively). Figure 1. shows the frequency of reasons why respondents found questions difficult to answer in the two WTP exercises.

### Willingness to pay for radiological image assessment

Nearly one-third of participants who originally chose to have their image assessed by a radiologist were unwilling to pay any money for a secondary expert opinion from an AI. Of those who chose AI, more than 22% were willing to pay to have their image analysed with the method of their preferred method. In this task, no one offered more than 559 EUR for any of the options. (Table 2.)

Willingness to pay was significantly lower for respondents choosing the radiologist compared to those who chose the AI to assess their image and make the diagnosis, however the effect size was small (Cohen’s D -0.181, 95% CI -0.292 - -0.070). In both subgroups, significant differences were also observed along educational level and income groups. (Table 3.; Online resource 2.)

At the question of whether it was difficult to give an answer in the radiological image assessment task, the level of disagreement was 34.0% and 42.8% in the subgroups choosing the radiologist or the AI to make the diagnosis, respectively. The proportion of those who neither agreed nor disagreed was 30.1% and 23.2%, respectively. (Online resource 3.)

Any difficulties with the task were reported by *N* = 614 and *N* = 269 respondents in the radiologist and AI subgroups, respectively. The most commonly reported difficulties were deciding on the amount to pay (45.9% and 46.5% in the two groups) and choosing between the two available methods (47.6% and 37.2% in the two groups). (Fig. 1.)

### Subsample indicating zero WTP in both tasks

In total, there were 341 (24.4%) people who did not want to pay money in any of the tasks. Their average age was 58.1 (SD = 9.9) years, 49.9% of them were female, and they had significantly (*p* = 0.004) lower average net income per capita compared to those who had a WTP greater than zero (379.7 EUR, SD = 235.7 vs. 426.6 EUR, SD = 239.6). Among them, more than 40% totally disagreed that it was difficult to answer the questions regarding the tasks, which was numerically higher compared to those who expressed their WTP (42.5% vs. 33.1% for hip replacement and 44.0% vs. 34.7% for the radiological image analysis task). Those who had zero WTP were less likely to report any problems with understanding the tasks than those who were willing to pay (57.5% vs. 66.9% for hip replacement and 56.0% vs. 65.3% for radiological image analysis task, respectively).

### Correlations between willingness to pay and background variables

The results of the Shapiro-Wilk test indicated that the continuous variables included in the analysis followed a non-normal distribution. In the total sample, the correlation of WTP with age and income was significant but weak in both the hip replacement surgery (*r* = 0.107, *p* < 0.001 and *r* = 0.162, *p* < 0.001, respectively) and radiological image assessment tasks (*r* = 0.093, *p* < 0.001 and *r* = 0.179, *p* < 0.001 respectively). With the eHEALS, the observed correlations were also weak and significant in case of radiological image assessment (*r* = 0.071, *p* = 0.008), but not for hip replacement surgery (*r* = 0.019, *p* = 0.481). There was no significant correlation between WTP and the EQ-5D-5 L index score in any of the tasks. With the EQ VAS, a significant but also weak correlation was found for the radiological image assessment (*r* = 0.088, *p* = 0.001), but not for the hip replacement surgery task (*r* = 0.046, *p* = 0.084).

### Regression analysis

The results of the regression analysis can be seen in Table 4. When controlling for respondent characteristics, WTP was lower if the preferred choice of intervention was robotic surgery than if the respondent preferred to be operated by a surgeon (Model 1). However, this relationship was not significant for radiological image assessment (Model 2). In both models, WTP was significantly higher if the respondent had a higher income, and this relationship was stronger for hip replacement surgery. Other factors significantly associated with WTP were age (Model 1), and eHEALS score (Model 2). Socio-demographic characteristics not associated with WTP in any of the tasks were sex, education, residency, employment, whether the respondent was in a relationship and whether the respondent lived with someone in the household. Similarly, the respondent’s health status as measured with the EQ-5D-5 L and whether the respondent had any implant were not significantly related to WTP. The level of reported difficulty in either task was also not associated.

**Table 4:** Results of the regression analysis

Variables	M1 (hip replacement)	M2 (radiological image assessment)
	Coefficients (SE)	
**Hip replacement (ref: surgeon)**		
Robot-assisted	-45.42 (22.29)*	-
**Radiological image assessment (ref: radiologist)**		
AI	-	11.67 (7.13)
**Sex (ref: female)**		
Male	-38.20 (22.46)	-2.27 (6.95)
**Age**	2.03 (0.81)*	0.32 (0.25)
**Education (ref: primary)**		
Secondary	-8.41 (27.11)	-2.59 (8.41)
Tertiary	24.69 (30.13)	9.28 (9.29)
**Health education (ref: No)**		
Yes	64.34 (43.08)	16.41 (13.31)
**Residency (ref: Capital)**		
Town	0.09 (27.03)	-2.02 (8.38)
Village	-58.36 (32.30)	-12.27 (10.00)
**Net income per capita**	0.28 (0.05)***	0.09 (0.02)***
**Paid work (ref: No)**		
Yes	-69.94 (41.39)	-21.30 (12.77)
**Living in a relationship (ref: No)**		
**Yes**	-20.29 (28.73)	0.06 (8.92)
**Living with someone in the household (ref: No)**		
Yes	62.22 (33.38)	11.52 (10.35)
**Any implant (ref: No)**		
Yes	-4.82 (22.72)	2.61 (7.04)
**eHEALS score**	1.19 (1.69)	1.32 (0.52)**
**EQ-5D-5 L index score**	-18.93 (40.55)	-2.91 (12.55)
**Difficulties in answering questions about hip replacement**	0.27 (5.47)	-
**Difficulties in answering questions about radiological imaging analysis**	-	-2.43 (1.69)
*No of observations*	*1165*	*1164*
*R* ^*2*^	*0.219*	*0.304*
*F-statistic*	20.14***	31.41***

* *p* < 0.05; ** *p* < 0.01; *** *p* < 0.001

The constant term was excluded from the analysis

## Discussion

The aim of this study was to assess social preferences for advanced digital health technologies using the hypothetical examples of robot-assisted (RA) hip replacement surgery and AI-based radiological image analysis for tumour assessment. Slightly more than half of the respondents (54.4%) opted for RA hip replacement surgery over conventional surgery, whilst a smaller proportion (33.6%) chose AI over radiologist for the image assessment. Significant difference was found between the two subgroups in both hypothetical situations along educational level (i.e., more educated respondents chose the digital technology) but income level was different only in the hip replacement task. In the WTP part, only about one-third would not have been willing to pay to be treated by their preferred method if they had been assigned to the opposite method for hip replacement surgery (surgeon: 32.8%, RA: 31.2%), and somewhat fewer respondents indicated zero WTP in the image analysis task (radiologist: 29.2%; AI: 22.5%). The regression analysis revealed significant association between WTP and respondents’ income level in both hypothetical situations. In addition, in the hip replacement task, WTP was higher if the respondent preferred the conventional surgery and increased with age. In the image evaluation task, a positive association was observed between WTP and eHEALS.

Compared to previous studies, our results showed a higher level of acceptance of RA surgery for hip replacement. In 2023, Abdelaal and colleagues (USA) found in a questionnaire survey among potential candidate patients for total knee arthroplasty that patients would primarily prefer the conventional surgery to the RA procedure, with just over 40% opting for the advanced technology [20]. The design and sampling of the study by Muaddi and colleagues (Canada, USA) was more similar to ours, nonetheless, just over a third of participants chose the RA procedure over laparoscopic surgery [42]. In contrast, we observed that more participants opted for the RA procedure in the hip replacement surgery task. It would be worth examining how technological, economic, cultural and healthcare system differences between countries influence the acceptability of and preferences for RA surgeries.

The preference for complex digital technology seems to depend on the type of medical procedure. In the radiological image evaluation task, the great majority, around two-thirds of the respondents, preferred a radiologist rather than an AI. This observation is consistent with the findings of Juravle and colleagues who reported in 2020 that participants have less confidence in a diagnosis made by an AI than a human physician [43]. Another possible explanation for the differences in acceptance could be that those who preferred the radiologist assessment over AI reported the most difficulties with understanding the description of the procedures. This may indicate that these respondents were likely to have less knowledge of the technology in general. However, it has been previously described that awareness does not correlate with the adoption of clinical AI [24]. We also found that those who chose the AI-based procedure were older on average than those who chose the radiologist. It was not the aim of our study to analyse factors influencing choice preferences among the elderly, however, it has been previously described in the literature that older people’s preferences for advanced technologies are shaped by a combination of factors such as technology concerns, expected benefits, available alternatives and social influences [44]. 

Educational level seems to play a key role in openness to digital health interventions, as those who chose the digital technology in both tasks had higher levels of education and e-health literacy. Other sociodemographic factors had variable importance in the two hypothetical situations. For instance, those who chose RA hip replacement had higher household income, while image evaluation was not associated with income. In terms of current health status, no difference was observed. Previous health experiences might have had an impact on preferences, however, this was only limitedly testable in our study. Since the study involved respondents from the general population, only a very small portion of respondents were expected to have direct experiences with the medical situations described in the tasks. Therefore, setting a cut-off point of 40 years for inclusion in the study was a deliberate step, partly in order to increase the proportion of respondents with personal experience of implantable medical devices (and apply the hypothetical situation of needing to have hip replacement among those who are closer to the typical age of this intervention). However, only 33 respondents had hip replacement surgery in the sample. On the other hand, RA surgery is not yet widely used in the clinical practice, therefore, the population lacks its own experiences in general. AI plays an increasing role in the image analyses, however the radiological diagnosis is established and signed by a radiologist. Studies focused on specific patient groups could provide a better understanding of the impact of past experiences on preferences.

In both WTP tasks, approximately one-third of the respondents were not willing to pay any money to secure their preferred procedure over the other option. This share is quite low compared to the results of Abdelaal and colleagues, who found in their survey that only less than one-tenth of participants were willing to pay for RA knee replacement surgery in a WTP task [20]. Respondents who were not willing to pay might be less likely to stick with their choices (for various reasons) and, therefore, have lower WTP for advanced digital technologies. Affordability is a common bias factor in WTP [45], however we find it important to highlight that there were options to pay really small amounts to decrease the influence of financial status on stated preferences. Another important aspect in this context is that previous studies have shown that preferences for health technologies are fundamentally influenced by their reimbursement scheme [46, 47]. However, in our study, we were not able to assess how WTP is related to reimbursement, as in the two hypothetical decision tasks in the survey, respondents were not provided with information about the financing scheme through which they would access the technology. Nevertheless, we believe that this could be the subject of future research, as knowledge of how these factors are associated with social preferences could help both financing bodies and health policymakers in decision-making about advanced digital health technologies.

According to the descriptive analysis, WTP amounts were very similar in the conventional and RA hip replacement surgery subgroups. However, although fewer participants chose the AI option in the second task, a higher WTP was observed for the AI-made secondary opinion compared to the radiologist. In the subgroup analysis, we observed that respondents with higher education and income had higher WTP for their preferred choices in all cases. We also observed a weak positive correlation between income and WTP. These results are consistent with those already reported in the literature regarding the WTP method. Education has been positively correlated with WTP, and income is also known to influence WTP, as people with higher incomes can afford to spend more [45]. 

WTP may be confounded by a number of factors that do not show any correlation in an univariate analysis [48]. Therefore, a multiple regression analysis was conducted to further analyse and understand the relationship between participants’ WTPs, preference for available methods, and background characteristics, including income. In contrast to the descriptive analysis, in the regression analysis, we found significantly higher WTP for those who opted for the conventional surgery. However, in the radiological image evaluation task, WTP was not significantly associated with the preferred method. In terms of patient characteristics, many studies have reported that participants’ gender, age, marital status, education, place of residence, employment and income are significant determinants of WTP for health services [48]. In contrast, we identified income alone as a factor associated with WTP in both tasks. Higher age was associated with higher WTP for the hip replacement surgery task, while the association between electronic health literacy and WTP was only significant for the radiological image assessment task. At the same time, no association was found along other patient characteristics, including those described in the literature as commonly correlated with WTP [48]. 

Limitations of our study need to be considered for the interpretation of the results. The hypothetical healthcare situations could be perceived differently by participants in terms of disease severity, possible outcomes and risks of interventions, general knowledge regarding RA and AI medical technologies. The difference may also be explained by the fact that the radiological image assessment process is conducted hiddenly from the patient in the background. Surgical intervention, on the other hand, is a more concrete activity of which the patient might have direct experience, either from his or her own medical history or from that of relatives and acquaintances. Assessment of these factors would be valuable to better understand the results and the reasons why fewer participants opted for the AI-made radiological image analysis and diagnosis compared to the RA hip surgery. Another limitation is that WTP is likely to be influenced by the design itself, thus many factors need to be considered, such as the severity of the situation described. Since we used the stated preference method to explore preferences, real word choices might be different. A further limitation is that the sampling frame of the survey was treated as confidential information, and therefore, the authors had no information on the response rate. However, we believe that this does not affect the reliability of the results due to the large sample size and the quota sampling used to ensure representativeness in terms of sex, age, education and type of residence. We used a cut-off point of 40 years as inclusion criteria, hence our study does not provide evidence on the preferences of younger adults. A limitation related to the analysis was that the continuous variables included were not normally distributed. However, to the best of our knowledge, the violation of this assumption has only little or no effect on the validity of the results, especially given the large sample size of the study [49–52]. In addition, we also acknowledge that, despite their significance, the correlation coefficients observed in the study were small, indicating a weak association of WTP with age, income, eHEALS and the EQ VAS. In addition, the R-squared values in the regression analysis were also low. We believe this is due to the complex and multifactorial nature of WTP, which is influenced by a number of factors. In line with this, previous studies published in the literature have also found weak associations and low R-squared values in linear regression analyses similar to our study [48]. 

Nonetheless, our research has a number of strengths as well. It was conducted on a large sample, representative of the general adult population of Hungary, thereby filling an important knowledge gap regarding social preferences on RA and AI-based medical technologies. We tested two different advanced digital technologies in parallel, which allowed a better understanding of the contextual dependency of preferences. Our study offers insight into the relationship between individuals’ self-reported electronic health literacy and their views on RA and AI medical technologies, which has been an underexplored area so far. For future research, we suggest investigating the generalisability of our findings with respect to other RA or AI-driven medical technologies, validating our results with other valuation methods, exploring the determinants of preferences further (e.g., impact of reimbursement context, free choice of physician, age-dependency of choices and WTP) and comparing hypothetical and real-world choices in specific patient groups. We encourage pre-post studies to assess the effects of eHealth-targeted public health campaigns on preferences of AI-based technologies, and re-evaluation of our findings in new studies when the penetration of AI technologies has become higher in healthcare.

## Conclusions

The results of this study suggest that there is considerable societal openness towards advanced robot-assisted and AI-based health technologies. A large number of participants expressed their desire to use them, who tended to be older and have higher income. However, no clear differences were observed in the strength of preferences as measured by the WTP method. Our results suggest correlation between the WTP and both the education and the income levels, although the multiple regression analysis revealed a clear relationship between WTP and income only. The regression also showed that respondents who opted for the conventional surgery had higher WTP, but no difference was observed for the radiological image assessment. Our findings are of considerable value to clinicians involved in the provision of care, who can gain insights into societal attitudes towards and acceptance of the emerging new advanced technologies, helping them to make therapeutic decisions and design clinical interventions. Technology developers may also benefit from the observations of this research, as knowledge of social preferences is essential in determining the direction of technology development. Prospective studies are encouraged in the future to better understand how individual factors influence WTP and to investigate their causal relationship.

### Electronic supplementary material

Below is the link to the electronic supplementary material.

Online Resource 1

Online Resource 2

Online Resource 3

## Data Availability

The data supporting the results of the analysis are available on request from the corresponding author.

## List of Abbreviations

WTP: willingness to pay
AI: artificial intelligence
RA: robot–assisted
eHEALS: electronic health literacy scale
