# Supplementary material for: Robot-assisted surgery and artificial intelligence-based tumour diagnostics: social preferences with a representative cross-sectional survey
Source: BMC Med Inform Decis Mak. 2024 Mar 27;24:87. doi: 10.1186/s12911-024-02470-x (PMC10981282; doi:10.1186/s12911-024-02470-x)
Supplement: Supplementary file 1 — Online Resource 1 [file 12911_2024_2470_MOESM1_ESM.docx]

**Online resource 1.**

**Survey questions**

**Intr1. If you agree, please put a 'tick' in the cells next to the statements.**

 1. I declare that I have read and understood the prospectus for the study, dated 16 May 2021.

 2. I acknowledge that my participation is entirely voluntary, that my consent is given without influence and that I have the right to withdraw at any time for any reason.

 I understand that the results of the study may be used in presentations or published in scientific reports, but that my identity will never be revealed.

 4. I agree to participate in the above mentioned study.

A1. Your gender:


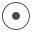
 1. Female


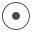
 2. Male

A2. When were you born?

Please enter the year!

1. .................................................................year

A8. Please select your place of residence from the drop-down menu.

When you start typing the name of the municipality, you can easily select the right one from the list that appears.


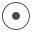
 1.

A3. What is your highest level of completed education?


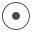
 1. Did not complete the first year of primary school


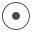
 2. Primary school Grades 1-3


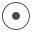
 3. Primary school, Grades 4-5


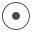
 4. Primary school Grades 6-7


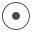
 5. Primary school Grade 8


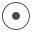
 6. High school, without matriculation and vocational diploma


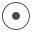
 7. High school, without matriculation, but with a vocational diploma


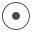
 8. High school with matriculation


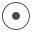
 9. University/College without diploma


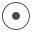
 10. University/College with diploma

A4. What is your marital status? Which category best describes your situation?


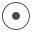
 1. Married


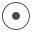
 2. Living in a civil partnership


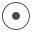
 3. Single


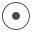
 4. Widowed


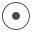
 5. Divorced


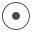
 6. Other

A5. Which of the following best describes your employment situation?

If more than one category applies to you (e.g. part-time worker and student), please choose the one that applies most of the time.


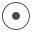
 1. I work full-time / I am self-employed


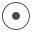
 2. I work part-time


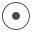
 3. I am a pensioner


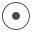
 4. I am a disability pensioner


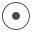
 5. I am a full-time student /I am a university student


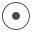
 6. I am unemployed (looking for a job)


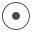
 7. I am unemployed (not looking for work)


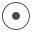
 8. I am a householder


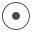
 9. Other

A6. Do you have a job that provides a regular income?


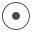
 1. Yes


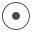
 2. No


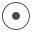
 3. I do not wish to answer

A7. Do you have a medical qualification?


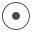
 1. Yes


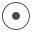
 2. No

**In the following, we would like to ask you a few questions about medical devices that are usually implanted in the body. These devices are also known as implants. There are many types of implants, such as cataract surgery eye lenses, pacemakers, hip replacements or breast implants inserted during breast plastic surgery. Their common feature is that they are implanted in the body for therapeutic purposes.**

**Some implants are inserted only temporarily (e.g. fixing a broken bone with a metal plate and removing it after healing; an IUD in the womb for contraception). Other implants are inserted into the body to remain there permanently (e.g. hip replacement).**

**We want to know if you have ever had an implant in your life. To do this, we have listed some of the most common types of implants per body region.**

B1. Please indicate if you have had any of the following types of implants.

You can mark more than one answer!

**Joints**

 1. hip replacement implantation

 2. knee replacement implantation

 3. device built into the spine

**Eye**

 4. cataract surgery (eye lens implantation)

**Breast**

 5. breast implantation (plastic surgery for breast augmentation or after cancer)

**Dentistry**

 6. insertion of dental implants (tooth implantation)

 7. dental bone replacement surgery

**Cardiology**

 8. implantation of a heart rhythm regulator (pacemaker)

 9. artificial heart valve

 10. coronary stenting (stent: a mesh-like, tubular device implanted in the veins of the heart to prevent blockage of the heart vessels)

**Abdomen**

 11. abdominal mesh implantation (due to abdominal hernia)

**Measuring instruments**

 12. subcutaneous blood glucose meter (sensor) for diabetics

**Other medical device (implant) implanted for a permanent purpose**

 13. other, namely:.................................................................

 14. other, namely:.................................................................

 15. other, namely:.................................................................

99. I do not have implants

**Now imagine that you have hip joint disease, which has severely limited your hip movement and is now a major hindrance to your daily activities.**

**Your doctor has recommended a hip replacement. Hip replacement surgery is a surgical procedure in which the hip joint is replaced with an implantable hip prosthesis made of artificial material (metal, plastic).**

E2a. We continue to ask you to imagine that you need hip replacement surgery for hip joint disease.

For hip replacement surgery, a surgical robot has been developed that is able to perform some phases of the operation completely autonomously.

In an emergency, the doctor can switch off the robot at any time and take over the operation. Both procedures are equally safe and produce the same results. If you had to decide, which would you choose?


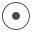
 ► 1. the doctor performs the operation in the traditional way, without the help of a surgical robot


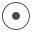
 ► 2. the doctor performs the operation using a surgical robot

E3a. You have been booked for surgery with the help of a surgical robot, but you have the option to have surgery without the help of a surgical robot for a fee. What is the maximum amount you would be willing to pay to have the surgery performed without the assistance of a surgical robot?

**Appears if: E2a == 1**


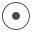
 1. 0 HUF


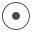
 2. 1 - 10 000 HUF


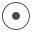
 3. 10 001 - 30 000 HUF


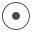
 4. 30 001 - 50 000 HUF


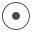
 5. 50 001 - 100 000 HUF


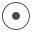
 6. 100 001 - 200 000 HUF


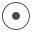
 7. 200 001 - 400 000 HUF


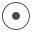
 8. 400 001 - 800 000 HUF


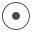
 9. More than 800 000 HUF : please specify the exact amount:.................................................................

E3b. You have been booked for surgery without the assistance of a surgical robot, but you have the option to have surgery performed with the assistance of a surgical robot for a fee. What would be the maximum amount you would be willing to pay to have the surgery performed with the help of a surgical robot?

**Appears if: E2a == 2**


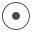
 1. 0 HUF


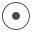
 2. 1 - 10 000 HUF


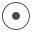
 3. 10 001 - 30 000 HUF


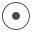
 4. 30 001 - 50 000 HUF


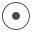
 5. 50 001 - 100 000 HUF


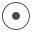
 6. 100 001 - 200 000 HUF


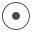
 7. 200 001 - 400 000 HUF


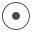
 8. 400 001 - 800 000 HUF


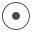
 9. More than 800 000 HUF : please specify the exact amount:.................................................................

E3c. To what extent do you agree with the following statement?

Questions about hip replacement surgery were difficult to answer.

|  | 1 - Totally agree | 2 | 3 | 4 - Neither agree nor disagree | 5 | 6 | 7 - Totally disagree |
| --- | --- | --- | --- | --- | --- | --- | --- |
| 1. | 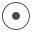1 | 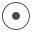2 | 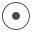3 | 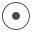4 | 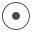5 | 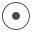6 | 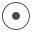7 |

E3d. Why did you find it difficult to answer the questions?

You can mark more than one answer!

**Appears if: E3c_1 == 1 || E3c_1 == 2 || E3c_1 == 3 || E3c_1 == 4 || E3c_1 == 5 || E3c_1 == 6**

 ► 1. I found it difficult to understand the situation caused by the hip disease.

 ► 2. I found it difficult to imagine the need for hip replacement surgery.

 ► 3. I found it difficult to understand the two surgical options.

 ► 4. I found it difficult to choose between the two surgical procedures.

 ► 5. I found it difficult to indicate the amount of money I would be willing to pay.

 6. Other:.................................................................

E4a. Imagine that you are undergoing a pre-operative imaging scan before the hip replacement surgery, where the images reveal a suspected tumour. Treatment depends on whether the tumour is benign or malignant. The following two options are available to assess the images. If you had to decide, which would you choose?


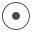
 ► 1. the images are assessed and diagnosed by a radiologist


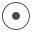
 ► 2. the images are analysed and diagnosed by artificial intelligence (artificial intelligence is a computer algorithm that has been trained to diagnose thousands of similar cases)

E5a. You have been analysed and diagnosed by an artificial intelligence, but you have the option to get a second opinion from a specialist radiologist for a fee. What would be the maximum amount you would be willing to pay to have a radiologist also perform an assessment?

**Appears if: E4a == 1**


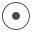
 1. 0 HUF


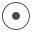
 2. 1 - 1000 HUF


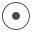
 3. 1001 - 5000 HUF


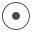
 4. 5001 - 15 000 HUF


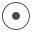
 5. 15 001 - 25 000 HUF


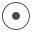
 6. 25 001 - 35 000 HUF


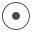
 7. 35 001 - 100 000 HUF


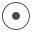
 8. 100 001 - 300 000 HUF


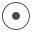
 9. more than 300 000 HUF: please specify the exact amount:.................................................................

E5b. You have been assessed and diagnosed by a radiologist, but you can also get a second opinion for a fee using artificial intelligence. What would be the maximum amount you would be willing to pay to have an AI assessment performed?

**Appears if: E4a == 2**


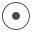
 1. 0 HUF


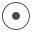
 2. 1 - 1000 HUF


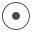
 3. 1001 - 5000 HUF


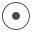
 4. 5001 - 15 000 HUF


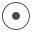
 5. 15 001 - 25 000 HUF


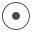
 6. 25 001 - 35 000 HUF


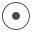
 7. 35 001 - 100 000 HUF


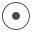
 8. 100 001 - 300 000 HUF


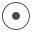
 9. more than 300 000 HUF: please specify the exact amount:.................................................................

E5c. To what extent do you agree with the following statement?

Questions about hip replacement surgery were difficult to answer.

|  | 1 - Totally agree | 2 | 3 | 4 - Neither agree nor disagree | 5 | 6 | 7 - Totally disagree |
| --- | --- | --- | --- | --- | --- | --- | --- |
| 1. | 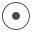1 | 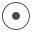2 | 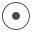3 | 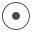4 | 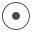5 | 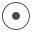6 | 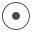7 |

E5d. Why did you find it difficult to answer the questions?

You can mark more than one answer!

**Appears if: E5c_1 == 1 || E5c_1 == 2 || E5c_1 == 3 || E5c_1 == 4 || E5c_1 == 5 || E5c_1 == 6**

 ► 1. I found it difficult to understand the situation regarding suspected cancer.

 ► 2. I found it difficult to imagine that I had a suspected cancer.

 ► 3. I found it difficult to understand the two possible ways of evaluating the images.

 ► 4. I found it difficult tp choose between two different evaluation methods.

 ► 5. I found it difficult to indicate the amount of money I would be willing to pay.

 6. Other:.................................................................

**With the following questions, we would like to know your views and experiences of finding health information online. For each question, please tick the answer that best applies to you at the moment.**

In this part of the survey, the eHEALS questionnaire^[[1]](#footnote-1)^ was used, for which the Hungarian version was developed and validated by Zrubka and colleagues.^[[2]](#footnote-2)^

**The following questions are designed to get a better understanding of your current situation.**

**In this part of the survey, the EQ-5D-5L was used in its original form^[[3]](#footnote-3)^, for which the Hungarian version was developed and officially accepted by the EuroQoL. For details please visit the EuroQol website (**[**https://euroqol.org/**](https://euroqol.org/)**).**

**Finally, we would like to ask you some questions about where you live and your household.**

I2. The following questions apply to your household.

Including yourself, how many people live in your household?

1. .................................................................18 or older

2. ................................................................. Person under 18 years of age

I3. What is your household's monthly net income?

Please include all income from work, business, rental property and investments, pensions, family or child support, unemployment benefits or other social assistance received by any member of the household.


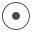
 1. 0 - 50 000 HUF


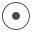
 2. 50 001 - 100 000 HUF


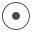
 3. 100 001 - 150 000 HUF


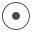
 4. 150 001 - 200 000 HUF


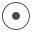
 5. 200 001 - 250 000 HUF


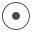
 6. 250 001 - 300 000 HUF


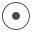
 7. 300 001 - 350 000 HUF


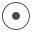
 8. 350 001 - 400 000 HUF


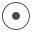
 9. 400 001 - 450 000 HUF


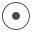
 10. 450 001 - 500 000 HUF


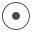
 11. More than 500 000 HUF


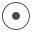
 12. I don't know


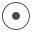
 13. I do not wish to answer

**Thank you for completing the questionnaire!**

1. Norman CD, Skinner HA. eHEALS: The eHealth Literacy Scale. J Med Internet Res. 2006 Nov 14;8(4):e27. doi: 10.2196/jmir.8.4.e27. [↑](#footnote-ref-1)
2. Zrubka Z, Hajdu O, Rencz F, Baji P, Gulácsi L, Péntek M. Psychometric properties of the Hungarian version of the eHealth Literacy Scale. Eur J Health Econ. 2019 Jun;20(Suppl 1):57-69. doi: 10.1007/s10198-019-01062-1. [↑](#footnote-ref-2)
3. Herdman M, Gudex C, Lloyd A, Janssen M, Kind P, Parkin D, Bonsel G, Badia X. Development and preliminary testing of the new five-level version of EQ-5D (EQ-5D-5L). Qual Life Res. 2011 Dec;20(10):1727-36. doi: 10.1007/s11136-011-9903-x. [↑](#footnote-ref-3)
