# Supplementary figures and images for: Robot-assisted surgery and artificial intelligence-based tumour diagnostics: social preferences with a representative cross-sectional survey
Source: BMC Med Inform Decis Mak. 2024 Mar 27;24:87. doi: 10.1186/s12911-024-02470-x (PMC10981282; doi:10.1186/s12911-024-02470-x)

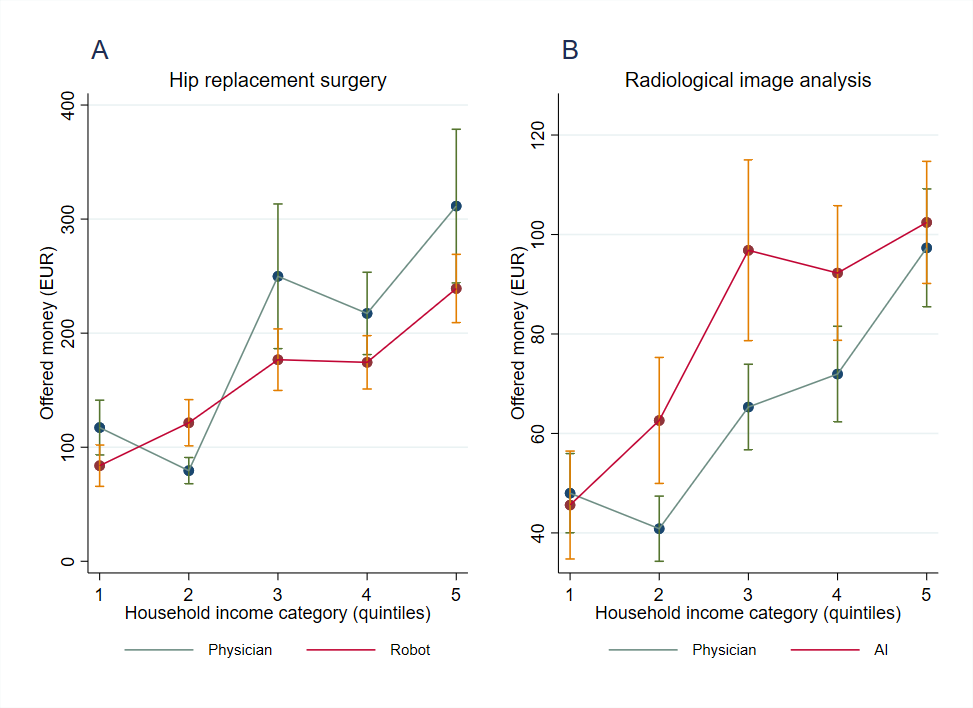


**Online resource 2. Participants’ willingness to pay by their household income (averages with 95% CIs)**

Supplement: Supplementary file 2 — Online Resource 2 [file 12911_2024_2470_MOESM2_ESM.docx]
