# Supplementary material for: Robot-assisted surgery and artificial intelligence-based tumour diagnostics: social preferences with a representative cross-sectional survey
Source: BMC Med Inform Decis Mak. 2024 Mar 27;24:87. doi: 10.1186/s12911-024-02470-x (PMC10981282; doi:10.1186/s12911-024-02470-x)
Supplement: Supplementary file 3 — Online Resource 3 [file 12911_2024_2470_MOESM3_ESM.docx]

**Online resource 3. Respondents’ agreement level with ’It was difficult to answer the questions regarding hip surgery and radiological image analysis’ statements after having completed the tasks**

|  | **Total sample** | **Preferred method**  **of surgery** | | **Preferred method for the**  **radiological image analysis** | |
| --- | --- | --- | --- | --- | --- |
|  |  | **Surgeon** | **Robot-assisted** | **Radiologist** | **AI** |
| Total number | 1400 (100.0) | 637 (100.0) | 762 (100.0) | 930 (100.0) | 470 (100.0) |
|  |  | **p<0.001** | | **p=0.001** | |
| Totally agree | 124 (8.9) | 64 (10.1) | 60 (7.9) | 79 (8.5) | 41 (8.7 ) |
| 2 | 57 (4.1) | 21 (3.3) | 36 (4.7) | 39 (4.2) | 12 (2.6) |
| 3 | 79 (5.6) | 42 (6.6) | 37 (4.9) | 71 (7.6) | 25 (5.3) |
| Neither agree nor disagree | 386 (27.6) | 213 (33.4) | 173 (22.7) | 280 (30.1) | 109 (23.2) |
| 5 | 94 (6.7) | 34 (5.3) | 60 (7.9) | 56 (6.0) | 43 (9.2) |
| 6 | 165 (11.8) | 63 (9.9) | 101 (13.3) | 89 (9.6) | 39 (8.3) |
| Totally disagree | 495 (35.4) | 200 (31.4) | 295 (38.7) | 316 (34.0) | 201 (42.8) |

Differences were tested with Kruskal-Wallis test.
